# Zona ophtalmique à localisation palpébrale: bonne évolution sous valaciclovir

**DOI:** 10.11604/pamj.2014.17.167.3887

**Published:** 2014-03-06

**Authors:** Fatim-Ezzohra Benotmane, Salim Belhassan

**Affiliations:** 1 Université Mohammed V Souissi, Service d'Ophtalmologie A de l'Hôpital des Spécialités, Centre Hospitalier Universitaire, Rabat, Maroc

**Keywords:** Zona ophtalmique, paupiére, valaciclovir, ophthalmic zoster, eyelid, valaciclovir

## Image en medicine

Je traite dans cette image le cas d'une patiente suivie dans notre formation, âgée de 55 ans sans antécédents pathologiques notables qui consulte aux urgences pour douleur, éruption cutanée au niveau de l'hémifront droit et la paupière supérieure non prurigineuse avec larmoiement et photophobie depuis une semaine. L'examen clinique trouve un œdème palpébral important unilatéral droit avec des lésions dermatologique d’âge différent type vésicule, pustule et quelques croutes qui occupent le territoire du nerf trijumeau (V1 ophtalmique) (A). L'examen ophtalmologique trouve une acuité visuelle conservée, pas de paralysie oculomotrice, une cornée claire avec test à la fluorescéine négative, une chambre antérieure optiquement vide, iris de trame et coloration normale. Le diagnostic de zona ophtalmique a été retenu devant ce tableau clinique; on amis la patiente sous 3g de valaciclovir par voie orale pendant 7 jours et un antalgique palier II, l’évolution a été marquée par la régression des lésions dermatologiques, la diminution de l’œdème et la douleur, par contre la malade a gardé des cicatrices dépigmentées (B). Le zona ophtalmique est une réactivation du virus de la varicelle et du zona (vzv) resté latent dans le ganglion de Gasser, le traitement antiviral doit être donné à la phase aigüe de la maladie pour diminuer la douleur en phase aigüe, la fréquence des algies post-zostériennes et les complications oculaires.

**Figure 1:**
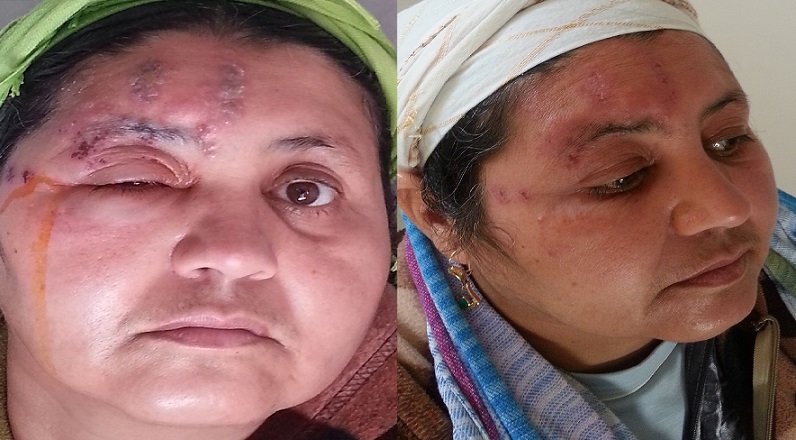
A) Montre les lésions dermatologiques du zona ophtalmique; B) Montre la régression des lésions dermatologiques sous 3g de valaciclovir pendant 7 jours laissant place à des cicatrices dépigmentées

